# Robot-assisted versus frame-based stereoelectroencephalography (sEEG) electrode implantation in drug-resistant epilepsy: a meta-analysis of accuracy, efficiency, and safety

**DOI:** 10.1007/s00701-026-06787-6

**Published:** 2026-02-21

**Authors:** Abdallah Abbas, Haneen Sabet, Karima El Refaei, Abrar AbuHamdia, Toka Elboraay, Yasmin Negida, Majed Aldehri, Ibrahim Alnaami, Ahmed M. Raslan

**Affiliations:** 1 Faculty of Medicine, Al-Azhar University, Damietta, Egypt; 2 Faculty of Medicine, South Valley University, Qena, Egypt; 3 School of Medicine, Newgiza University (NGU), Giza, Egypt; 4 Hamad Bin Khalifa University, Doha, 34110 Qatar; 5 Faculty of Medicine, Zagazig University, Zagazig, Egypt; 6 Department of Anatomy, College of Medicine, King Khalid University, 8082, 62523 Abha, Saudi Arabia; 7 Department of Neurosurgery, Mental Health and Neuroscience, Maastricht University Medical Center, Maastricht, Netherlands; 8 Division of Neurosurgery, Department of Surgery, King Khalid University, Abha, Saudi Arabia; 9 Department of Neurological Surgery, Oregon Health & Science University, Portland, OR USA

**Keywords:** Epilepsy, Frame-based, Robot-assisted, Stereoelectroencephalography, SEEG

## Abstract

**Objective:**

To compare the accuracy, efficiency, and safety of robot-assisted versus frame-based stereoelectroencephalography (sEEG) in patients with drug-resistant epilepsy.

**Methods:**

In accordance with the PRISMA guidelines, a comprehensive literature search was conducted across four databases (PubMed, Scopus, Web of Science, and Cochrane) up to September 2025. We included comparative studies that evaluated robot-assisted versus frame-based sEEG in patients with drug-resistant epilepsy. A random-effects model was applied to calculate the mean difference (MD) and risk ratio (RR) with corresponding 95% confidence intervals (CI).

**Results:**

Eight retrospective comparative cohort studies (758 patients) were included. Regarding accuracy, there was no significant difference between the robot-assisted and frame-based sEEG in depth error (MD: 0.24 mm; 95% CI: -0.79 to 1.27), radial error (MD: 0.07 mm; 95% CI: -0.70 to 0.84), entry point error (EPE; MD: -1.35 mm; 95% CI: -2.74 to 0.04), and target point error (MD: -0.02 mm; 95% CI: -0.57 to 0.53). Robot-assisted sEEG demonstrated a significantly shorter overall operation time (MD: -32.58 min; 95% CI: -47.92 to -17.24) and operation time per electrode (MD: -6.55 min; 95% CI: -8.08 to -5.02). However, pre-implantation time (MD: -1.46 min; 95% CI: -14.02 to 11.11) and electrode number per patient (MD: 0.86; 95% CI: -0.84 to 2.56) were comparable between groups. There was no significant difference between the two groups in overall complication rates, including hemorrhagic events, neurological deficits, infections, and technical complications.

**Conclusion:**

Robot-assisted sEEG significantly reduced both overall operation time and operation time per electrode compared with the frame-based group. Both techniques demonstrated comparable accuracy and safety profiles.

**Supplementary Information:**

The online version contains supplementary material available at 10.1007/s00701-026-06787-6.

## Introduction

Epilepsy is one of the most common chronic neurological disorders, affecting approximately 65 million people worldwide. In China, the lifetime prevalence of epilepsy was reported to be 0.457% in 2015 [38]. In 2021, the prevalence of active epilepsy was 1.1% (about 2,865,000 persons) and inactive epilepsy was 0.6% (roughly 1,637,000 adults) among adults in the United States [24].

Although antiseizure medications (ASMs) remain the first-line treatment, approximately 30% to 40% of patients exhibit inadequate seizure control [40]. For patients with focal drug-resistant epilepsy, surgical intervention is often the most effective option for achieving seizure freedom and improving quality of life [41]. Successful epilepsy surgery critically depends on accurately identifying the epileptogenic zone (EZ), the brain region whose resection results in seizure control. When non-invasive modalities such as video electroencephalography (EEG), magnetic resonance imaging (MRI), and positron emission tomography (PET) fail to localize the EZ, intracranial electrode implantation becomes necessary to directly record epileptic activity and map the epileptogenic network.

Stereoelectroencephalography (sEEG), first introduced by Talairach and colleagues in the 1950s [37, 39], is an invasive presurgical diagnostic technique that involves stereotactic depth-electrode implantation for intracranial EEG recording, allowing precise localization of the EZ [37]. This approach allows direct recording from both superficial and deep brain regions, including structures that are difficult to access using subdural grids, such as the insula and cingulate gyri [44]. sEEG has long been used in Europe for invasive delineation of the EZ in both children and adults [11, 40]. Traditionally, sEEG electrode implantation relied on Frame-Based Stereotaxy (FRS), a well-established technique for precise three-dimensional procedures. Historically regarded as a gold standard for spatial accuracy, FRS provides reliable guidance for electrode placement. However, it has inherent limitations: restricted trajectory planning (limited to linear or orthogonal paths), lengthy setup and calculations, and ergonomic challenges may arise during electrode insertion and manipulation [7, 8, 19, 25, 26, 28, 31].

Although the conceptual foundation of sEEG has remained largely unchanged, technological innovations in neuroimaging, stereotactic navigation, and robotic assistance have markedly improved its precision and safety for localizing the EZ, leading to its widespread adoption at epilepsy centers worldwide, progressively replacing subdural grid techniques in many epilepsy centers, including across China at epilepsy centers [14]. With the limitations of FRS and the increasing demand for higher precision and efficiency, robot-assisted systems (RAS) have emerged as the next major evolution in stereotactic neurosurgery.

The integration of robotic systems such as ROSA (Medtech/Zimmer Biomet) and Neuromate (Renishaw) into stereotactic surgery represents a major evolution in stereotactic neurosurgery. These RAS offer several theoretical advantages, including greater planning flexibility that allows complex, nonlinear trajectories; reduced operative time and faster workflow; and the maintenance of submillimetric accuracy comparable to or exceeding traditional frame-based techniques. In practical terms, RAS offers several advantages over manual or frame-based approaches, including the ability to plan oblique trajectories unrestricted by orthogonal coordinates and to generate accurate three-dimensional (3D) reconstructions of electrode paths [10, 12, 13, 18, 21, 29, 30].

Moreover, robot-assisted implantation has demonstrated favorable safety profiles, with complication rates comparable to or lower than those of frame-based approaches [14]. RAS has also expanded the accessibility of sEEG, even in institutions lacking traditional frame-based systems such as those of Talairach or Leksel. Consequently, RAS is now increasingly utilized across pediatric and adult epilepsy centers for more accuracy, efficiency, and safety in the localization of the EZ. Despite these advances, few studies have systematically compared robot-assisted sEEG with the classical Talairach frame-guided method [6, 9].

This systematic review and meta-analysis aims to compare robot-assisted versus frame-based sEEG in patients with drug-resistant epilepsy, focusing on three major outcomes: accuracy of electrode placement, procedural efficiency (operative time), and safety (complication rates). By synthesizing evidence from available studies, we sought to clarify the relative advantages of both techniques and provide evidence-based guidance for clinical decision-making in epilepsy surgery.

## Methods

This meta-analysis was conducted in accordance with the PRISMA guidelines and the recommendations outlined in the *Cochrane Handbook for Systematic Reviews of Interventions* [15, 34]. The protocol was registered in PROSPERO (CRD420251001014).

### Search strategy & eligibility criteria

A systematic literature search was performed in Scopus, Web of Science, PubMed, and Cochrane CENTRAL from database inception to September 1, 2025. The following search query was applied: (("stereo-electro-encephalography" OR "stereo-electroencephalography" OR "stereoelectroencephalography" OR "SEEG") AND ("robot-assisted" OR "robotic" OR "robot*" OR "ROSA" OR "robot-guided" OR "frame*" OR "frame-based")).

The inclusion criteria for our meta-analysis were patients with epilepsy undergoing sEEG in studies that compared robot-assisted sEEG implantation with frame-based implantation in terms of accuracy, efficiency, and safety. Eligible studies were required to report at least one outcome. We included all comparative observational studies and clinical trials that met these criteria.

### Screening & data extraction

Screening and data extraction were performed independently by two authors, with any disagreements resolved through consultation with a third reviewer. Screening was conducted in two stages: an initial title and abstract screening, followed by a full-text screening of potentially eligible studies. Moreover, we screened references of included studies.

Data extraction included summary information such as study design, recruitment period, country, and details of the sEEG intervention, including robot or frame type, patient positioning, postoperative imaging, and electrode diameter. Baseline data were also extracted, including sample size, patient age at the time of sEEG, sex, preoperative MRI findings, and implantation site.

### Outcome measures

Outcomes included radial error in millimeters (mm), depth error (mm), entry point error (EPE) in mm, target point error (TPE) in mm, overall operation duration in minutes, operation duration per electrode in minutes (operation time ÷ electrode number), pre-implantation time in minutes, number of electrodes per patient, or complications.

Intracranial hemorrhage was defined as any postoperative radiologically detected bleeding (including intraparenchymal, subdural, epidural, or subarachnoid hemorrhage). Intracranial hematoma was defined as a space-occupying blood collection explicitly reported as a hematoma by the original study authors. Because these outcomes were inconsistently reported and not interchangeable across studies, they were analyzed separately.

### Bias risk assessment

The risk of bias for observational studies was assessed using the Newcastle–Ottawa Scale (NOS) [32]. This tool evaluates studies across three domains: selection of study groups, comparability of groups, and ascertainment of exposure and outcomes. Details regarding the scoring algorithm and specific domains are provided in the footnote of Supplementary Table 1.

### Statistical analysis

All statistical analyses were performed using Review Manager (RevMan) version 5.4 and R version 4.5.1 [35, 36]. Continuous outcomes were analyzed using mean difference (MD) with 95% confidence intervals (CIs), while categorical outcomes were analyzed using risk ratios (RRs) with 95% CIs. We utilized the Meta-Analysis Accelerator tool for statistical conversions [4]. A random-effects model was employed over a fixed-effect model, as it accounts for both within-study and between-study variability and provides more balanced weighting across studies with different sample sizes. Statistical significance was defined as a *p*-value < 0.05.

Operation time definitions varied across studies. To address this heterogeneity, operative duration was categorized into subgroups based on the reported definition, including skin-to-skin surgical time (from first incision to closure), frame-related procedural time (from frame installation to frame removal), and studies with not reported (NR) definitions.

Intracranial hemorrhage was analyzed as a composite outcome including intraparenchymal, subdural, epidural, and subarachnoid hemorrhage. These subtypes were grouped together to increase statistical power and ensure a methodologically sound comparison between robot-assisted and frame-based sEEG.

Heterogeneity was assessed using the chi-square test and quantified with the I^2^ statistic, with values of I^2^ > 50% and a chi-square *p*-value < 0.10 considered indicative of substantial heterogeneity [1]. In cases of significant heterogeneity, leave-one-out sensitivity analyses were performed to determine the influence of individual studies on the pooled effect estimates [2]. Given the number of outcomes assessed, we evaluated the robustness of the primary efficiency outcomes (overall operation time and number of electrodes per patient) using a conservative Bonferroni correction (α ≈ 0.0025). Doi plots and the Luis Furuya-Kanamori (LFK) index [8] were used to analyze publication bias and small-study effects when the outcome comprised five or more studies. Symmetry and minimal bias were indicated by LFK index values between − 1 and + 1, minor asymmetry was suggested by values between − 2 and − 1 or + 1 and + 2, and substantial asymmetry was indicated by values above ± 2. Because there are few studies available for the majority of outcomes, this approach was selected over Egger's test [3, 26].

## Results

### Search and screening

**Fig. 1:**
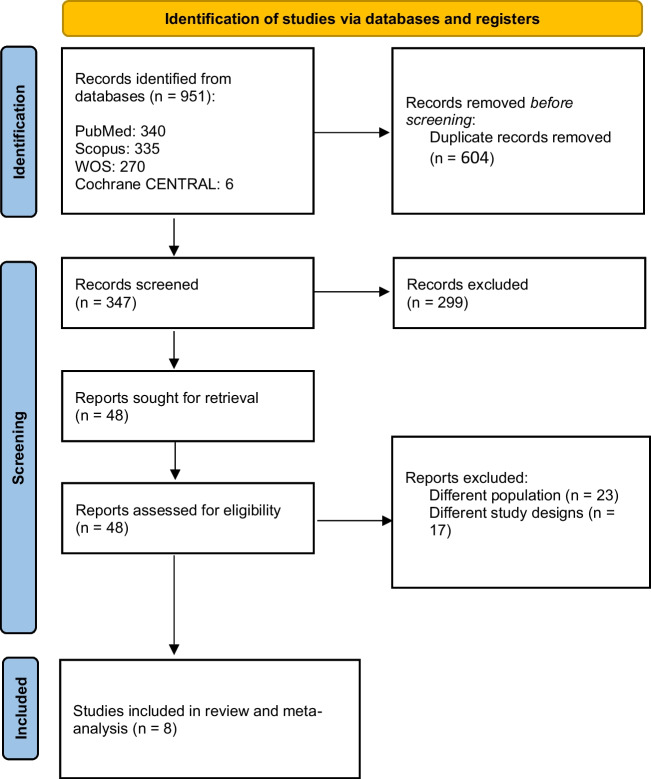
PRISMA flow diagram of study selection. Flowchart summarizing the screening and selection of studies included in the meta-analysis. Of 951 records initially identified, 604 duplicates were removed. After title/abstract and full-text screening, 8 studies were included in the final quantitative synthesis

After searching databases, 951 studies were identified, and 604 duplicates were removed. Title and abstract screening identified 48 studies eligible for full-text screening. After the full-text screening, eight studies were included in the final analysis [5, 6, 9, 20, 22, 27, 46, 47]. A detailed selection process is summarized in Fig. 1.

### Characteristics of the included studies

Eight retrospective cohort studies (758 patients) with recruitment durations ranging from 2000 to 2024 were included. Two of the studies were conducted in China, two in France, and others in locations like Canada, Australia, Germany, and Taiwan.

The robot-assisted sEEG group included a total of 310 patients (male: 52.58%) with a pooled weighted mean age of 26.85 (SD: 13.17). In the frame-based sEEG group, 448 patients were included (male: 52.68%) with a pooled weighted mean age of 26.29 (SD: 11.04).

The Leksell Frame type was used in three studies, and the Talairach frame was used in two studies. ROSA (Zimmer Biomet) was the most common robot used (3 studies), followed by both SINO and Neuromate Robot (two studies each). For additional details, please refer to Tables 1 and 2.

**Table 1:** Summary of the included studies

Study ID	Study design	Recruitment duration	Country	Definition of operation time	Frame-based sEEG				Robot-assisted sEEG				Summary of the study
					Frame type	Patient position	Post-operative imaging	Electrode diameters	Robot type	Patient position	Post-operative imaging	Electrode diameters	
Abdallat 2025	RC	2000 to 2020	Canada	Skin-to-skin surgical time (from first incision to closure)	Leksell Frame	NA	CT scan	10-contact, 3–6 mm spacing, (0.86 mm diameter)	Neuromate stereotactic (Neuroinspire planning software product version 4.2.12113.1; Renishaw plc)	NA	CT scan	10-contact, 3–6 mm spacing, (0.86 mm diameter)	This single-centre study found that robot-assisted sEEG significantly reduced operative time compared with the frame-based method
Fish 2025	RC	2019 to 2024	Australia	-	NA	Head secured in the CRW stereotactic head frame	Non-contrast CT scan	5–18 contacts with a 0.8 mm diameter	NA	Head secured in CRW head frame and a bone screw (2.0 mm diameter, 8 mm length)	CT scan	5–18 contacts with a 0.8 mm diameter	This study showed that the Medtronic auto guide robot safely improved pre-implantation and implantation time per electrode in sEEG, though accuracy was lower compared to the frame-based method
Han 2024	RC	2014 to 2022	Taiwan	Skin-to-skin surgical time (from first incision to closure)	Leksell Frame	Semi-sitting position	High-resolution MRI and CT	NA	ROSA (Zimmer Biomet)	Supine	High-resolution MRI and CT	NA	sEEG is a safe and accurate technique for epilepsy surgery, with multimodal planning and robotic assistance further enhancing safety, efficiency, and accuracy
Yao 2022	RC	2015–2019	China	Skin-to-skin surgical time (from first incision to closure)	CRW stereotactic frame (Integra, USA)	NA	CT scan	0.8 diameter	Sinovation robotic system (Sinovation, Beijing, China)	Supine	CT scan	Sinovation electrodes (0.8 diameter)	Robot-guided sEEG demonstrated higher entry point accuracy, and efficiency, though implantation accuracy was influenced by skull thickness and electrode-skull angle
Machetanz 2021	RC	2017 to 2020	Germany	-	Radionics Brown-Roberts-Wells (BRW) Frame	Supine	High-resolution T1-weighted MRI. If MRI wasn't available, CT was done	NA	ROSA One, Zimmer Biomet; robot group	The patient was placed supine, and the patient’s head was fixed in a Mayfield skull clamp and coupled to the robot	High-resolution T1-weighted MRI. If MRI wasn't available, CT was done	NA	Implantation of insular electrodes via AOA or POA was safe and efficient without loss of accuracy despite longer trajectories, and outcomes were comparable between frame-based and robot-assisted sEEG
Zheng 2021	RC	2018–2019	China	Skin-to-skin surgical time (from first incision to closure)	Leksell frame (Elekta, Sweden)	Supine	CT scan	Sinovation, 0.8 mm diameter	SINO (Sinovation, Beijing, China)	Supine	CT scan	Sinovation, 0.8 mm diameter	Robot-assisted sEEG was significantly more efficient than frame-based sEEG while maintaining comparable safety and effectiveness
Bourdillon 2019	RC	2012–2017	France	Frame installation to frame removal	Talairach frame	NA	MRI performed within 24	Microdeep depth electrodes, 0.8 diameter	Neuromate robot (Renishaw PLC)	NA	MRI performed within 24	Microdeep depth electrodes, 0.8 diameter	Robotic sEEG with intraoperative 3D angiography provided superior accuracy to the Talairach approach and highlighted a potential safety advantage over MRI-based planning
Abel 2018	RC	NA	France	NA	Talairach frame	Supine position	CT scan	NA	ROSA (Zimmer Biomet)	Supine	CT scan	NA	Frameless robot-assisted sEEG was safe and effective in children, faster than the Talairach frame-based method, with equivalent safety and efficacy, and offered greater trajectory flexibility for improved epileptic network localization

*BRW* Brown-Roberts-Wells stereotactic frame, *CRW* Cosman-Roberts-Wells stereotactic frame, *CT* Computed tomography, *ID* Identifier (Study ID), *mm* millimetre, *MRI* Magnetic resonance imaging, *NA* Not answered, *RC* Retrospective cohort, *ROSA* Robotic stereotactic system (ROSA robot, Zimmer Biomet), *sEEG* Stereoelectroencephalography, *SINO* Sinovation (SINO) robotic system, *T1* T1-weighted (MRI sequence), *USA* United States of America

**Table 2:** Baseline of the included studies

Study ID	Sample size (No.)		Age at sEEG, years, mean (SD)		Sex, Males: Females No		Preoperative MRI findings, No. (%)		Site of implantation, No. (%)	
	Frame-based group	Robot-assisted group	Frame-based group	Robot-assisted group	Frame-based group	Robot-assisted group	Frame-based group	Robot-assisted group	Frame-based group	Robot-assisted group
Abdallat 2025	104	88	33.8 (11.1)	34.8 (13.3)	50:54	45:43	NA	NA	Bilateral: 46 (44.2%), unilateral: 58 (55.8%)	Bilateral: 56 (63.6%), unilateral: 32 (36.4%)
Fish 2025	50	8	36.7	33.8	20:30	5:3	NA	NA	Bilateral: 17 (34%), unilateral: 33 (66%)	Bilateral: 6 (66%), unilateral: 2 (34%)
Han 2024	140	26	25 (9.55)	24 (12.19)	79: 61	10:16	Lesional: 90 (64.2%), non-lesional: 50 (35%)	Lesional: 14 (53.8%), non-lesional: 12 (46%)	Bilateral: 37 (26.4%), unilateral: 103 (73.57%)	Bilateral: 1 (3.8%), unilateral: 25 (96.8%)
Yao 2022	60	87	21 (9.8)	22.8 (9.5)	35: 25	46:41	Lesional: 30 (50%), non-lesional: 30 (50%)	Lesional: 58 (66.7%), non-lesional:29 (33.3%)	Bilateral: 5 (8.3%), unilateral: 55 (91.7%)	Bilateral: 3 (3.4%), unilateral: 84 (96.6%)
Machetanz 2021	12	15	23.2 (17)	26.5 (15.7)	3:9	7:8	NA	NA	NA	NA
Zheng 2021	14	19	18.4 (7.7)	19.5 (11.4)	8:6	9:10	Lesional: 8 (57.1%), non-lesional: 6 (42.9%)	Lesional: 14 (73.7%), non-lesional (26.3%)	Bilateral: 1 (7.1%), unilateral: 13 (92.9%)	Bilateral: 3 (15.78%), unilateral: 16 (84.2%)
Bourdillon 2019	50	50	29 (1.4)	29.6 (11.2)	27: 23	34:16	NA	NA	Bilateral: 26 (52%), unilateral: 24 (48%)	Bilateral: 16 (32%), unilateral: 34 (68%)
Abel 2018	18	17	11.3 (2.75)	11.3 (3.91)	14:4	7:10	Lesional: 14 (77.7%), non-lesional:4 (22.2%)	Lesional: 10 (58.8%), non-lesional:7 (41.1%)	NA	NA

*F* Females, *M* Males, *MRI* Magnetic resonance imaging, *NA* Not applicable, *No.* Number, *SD* Standard deviation, *sEEG* Stereoelectroencephalography

### Risk of bias assessment

The NOS tool evaluated a total of eight cohort studies. Seven studies [5, 6, 9, 22, 27, 46, 47] were rated as good quality, while one study [20] was rated as poor quality due to lack of compatibility and representativeness of the exposed cohort (Supplementary Table 1).

### Analysis of accuracy outcomes

#### Depth error and radial error

**Fig. 2:**
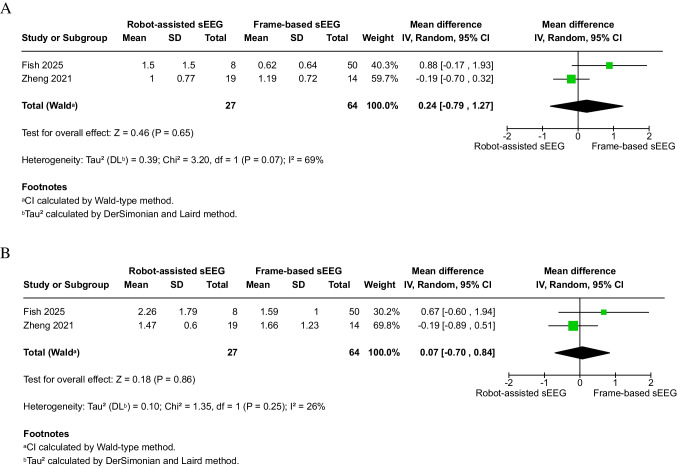

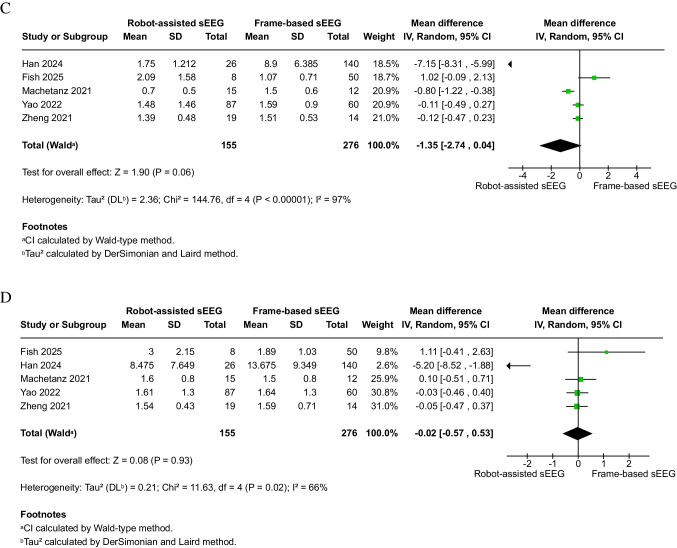
Accuracy outcomes of robot-assisted versus frame-based sEEG. **A** Meta-analysis of depth error (mm). **B** Meta-analysis of radial error (mm). **C** Meta-analysis of entry point error (EPE, mm). **D** Meta-analysis of target point error (TPE, mm). No statistically significant differences were found across accuracy measures, though heterogeneity varied by outcome. Sensitivity analyses are shown in Supplementary Figs. 1–2

There was no significant difference in depth error between robot-assisted (*n* = 27) and frame-based (*n* = 64) sEEG (2 studies, MD: 0.24 mm; 95% CI: − 0.79 to 1.27; *P* = 0.65) (Fig. 2A). Similarly, no significant difference was found in radial error (2 studies, MD: 0.07 mm; 95% CI: − 0.70 to 0.84; *P* = 0.86) (Fig. 2B). Heterogeneity was substantial for depth error (I^2^ = 69%, *P* = 0.07) but insignificant for radial error (I^2^ = 26%, *P* = 0.25).

#### EPE

No statistically significant difference was detected between robot-assisted (*n* = 155) and frame-based (*n* = 276) techniques (5 studies, MD: − 1.35 mm; 95% CI: − 2.74 to 0.04; *P* = 0.06) (Fig. 2C). However, heterogeneity was considerable (I^2^ = 97%, *P* < 0.00001). After excluding two studies (Han 2024 and Machetanz 2021) [22, 27], heterogeneity was reduced to an insignificant level (I^2^ = 47%, P = 0.15), and the results remained non-significant (Supplementary Fig. 1).

#### TPE

Similarly, there was no significant difference in TPE between robot-assisted (*n* = 155) and frame-based (*n* = 276) sEEG (5 studies, MD: − 0.02 mm; 95% CI: − 0.57 to 0.53; *P* = 0.93) (Fig. 2D). Heterogeneity was moderate to high (I^2^ = 66%, *P* = 0.02). Exclusion of Han 2024 [22] resolved heterogeneity (I^2^ = 0%, *P* = 0.53), but the overall results remained non-significant (Supplementary Fig. 2).

### Analysis of efficiency and operative outcomes

#### Overall operation time (minutes)

**Fig. 3:**
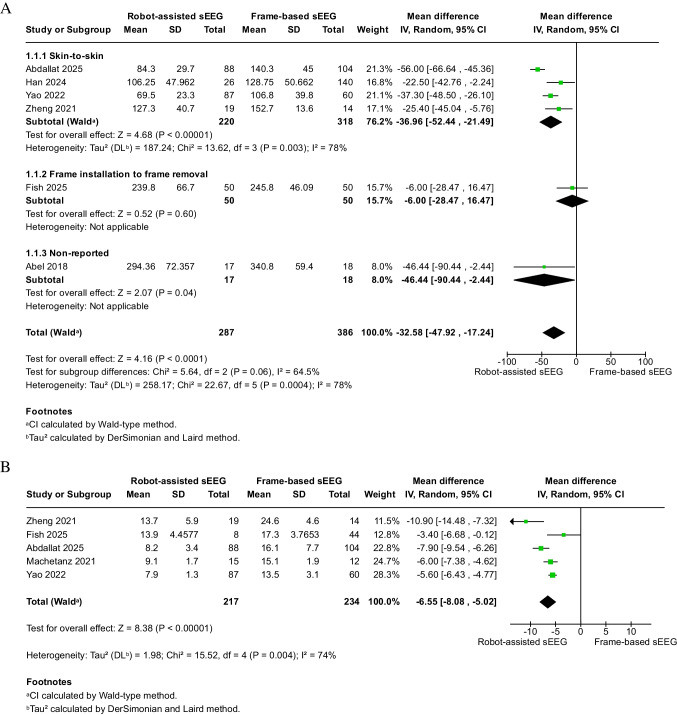

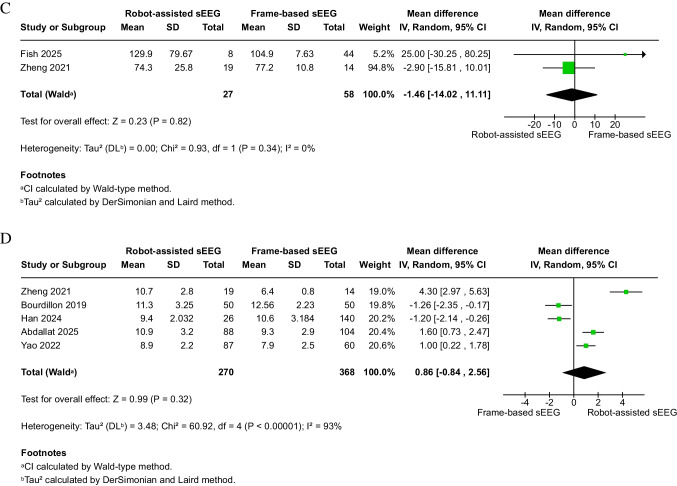
Efficiency outcomes of robot-assisted versus frame-based sEEG. **A** Overall operation duration (minutes). **B** Operation time per electrode (minutes). **C** Pre-implantation time (minutes). **D** Number of electrodes per patient. Robot-assisted implantation significantly reduced overall operation time and time per electrode compared with frame-based methods. No differences were detected for pre-implantation time or electrode number per patient. Sensitivity analyses are presented in Supplementary Figs. 3–4

Robot-assisted sEEG (*n* = 287) was associated with a significantly shorter overall operation time compared to frame-based sEEG (*n* = 386) (6 studies, MD: − 32.58 min; 95% CI: − 47.92 to − 17.24; *P* < 0.0001) (Fig. 3A). Subgroup analysis was conducted based on the definition; robot-assisted was associated with a decrease of 36.96 min in the skin-to-skin subgroup (four studies), 6 min in the frame installation to frame removal subgroup (one study), and 46.44 min in the not reported study.

Substantial heterogeneity was observed (I^2^ = 78%, *P* = 0.003) in the skin-to-skin subgroup. After omitting Abdallat 2025 [5], heterogeneity was reduced to a very low level (I^2^ = 6%, *P* = 0.34), and the results remained significant (MD: − 31.85 min; 95% CI: − 41.09 to − 22.60; *P* < 0.00001) (Supplementary Fig. 3).

#### Operation time per electrode (minutes)

Robot-assisted sEEG (*n* = 217) also demonstrated a significantly shorter operation time per electrode compared to frame-based sEEG (*n* = 234) (5 studies, MD: − 6.55 min; 95% CI: − 8.08 to − 5.02; *P* < 0.00001) (Fig. 3B). Considerable heterogeneity was detected (I^2^ = 74%, *P* = 0.004). After omitting Abdallat 2025 and Zheng 2021 [5, 47], heterogeneity was eliminated (I^2^ = 2%, *P* = 0.36), and the results remained robust (MD: − 5.60 min; 95% CI: − 6.32 to − 4.89; *P* < 0.00001) (Supplementary Fig. 4).

#### Pre-implantation time (minutes)

There was no significant difference in pre-implantation time between robot-assisted (*n* = 27) and frame-based (*n* = 58) sEEG (2 studies, MD: − 1.46 min; 95% CI: − 14.02 to 11.11; *P* = 0.82) (Fig. 3C). The data were homogeneous (I^2^ = 0%, *P* = 0.34).

#### Number of electrodes per patient

No significant difference was observed between robot-assisted (*n* = 270) and frame-based (*n* = 368) sEEG in terms of electrode number per patient (5 studies, MD: 0.86; 95% CI: − 0.84 to 2.56; *P* = 0.32) (Fig. 3D). Heterogeneity was considerable (I^2^ = 93%, *P* < 0.00001) and could not be resolved through leave-one-out analysis.

#### Bonferroni correction

Both overall operative time and operative time per electrode remained statistically significant after Bonferroni correction.

### Analysis of safety outcomes

**Fig. 4:**
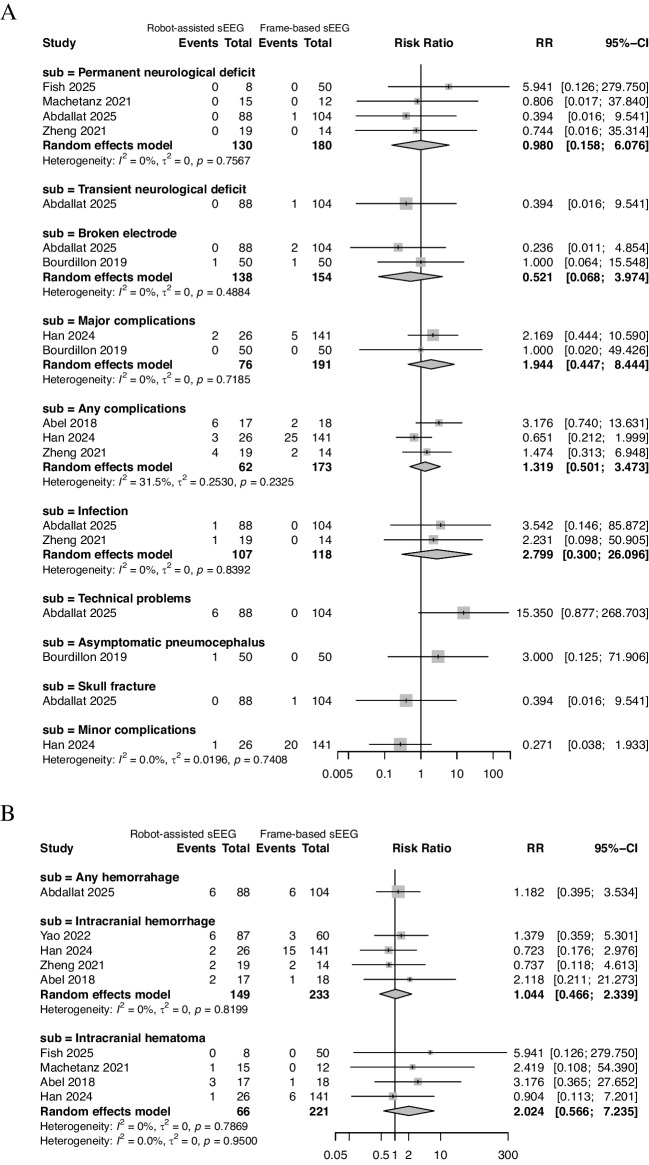
Safety outcomes of robot-assisted versus frame-based sEEG. **A** Pooled analysis of neurological complications, infections, technical problems, and other adverse events. **B** Pooled analysis of hemorrhagic complications. No significant differences in complication rates were observed between techniques

There was no significant difference between the robot-assisted and frame-based sEEG in terms of permanent neurological deficit (0%, 0/130 vs. 0.6%, 1/180), transient neurological deficit (0%, 0/88 vs. 1.0%, 1/104), broken electrode (0.7%, 1/138 vs. 1.9%, 3/154), major complications (2.6%, 2/76 vs. 2.6%, 5/191), any complications (21.0%, 13/62 vs. 16.8%, 29/173), infection (1.9%, 2/107 vs. 0%, 0/118), technical problems (6.8%, 6/88 vs. 0%, 0/104), asymptomatic pneumocephalus (2.0%, 1/50 vs. 0%, 0/50), skull fracture (0%, 0/88 vs. 1.0%, 1/104), and minor complications (3.8%, 1/26 vs. 14.2%, 20/141) (refer to Fig. 4A).

For hemorrhage, there was likewise no significant difference between robot-assisted and frame-based sEEG in terms of any hemorrhage (6.8%, 6/88 vs. 5.8%, 6/104), intracranial hemorrhage (8.1%, 12/149 vs. 9.0%, 21/233), and intracranial hematoma (7.6%, 5/66 vs. 3.2%, 7/221) (see Fig. 4B). There was no significant heterogeneity across the analysis of all these complications. Most included studies identified hemorrhagic events based on routine postoperative computed tomography (CT) or MRI. Differentiation between symptomatic and asymptomatic hemorrhages was inconsistently reported; therefore, the proportion of clinically symptomatic events could not be reliably determined.

### Publication bias assessment

Publication bias was assessed for EPE, TPE, overall operative time, and number of electrodes per patient using the LFK index derived from Doi plots (Supplementary Fig. 5). Minor asymmetry was observed for EPE (LFK = − 1.81) and TPE (LFK = − 1.53). In contrast, major asymmetry was detected for overall operative time (LFK = − 2.37), whereas no asymmetry was identified for the number of electrodes per patient (LFK = 0).

## Discussion

Our meta-analysis included eight retrospective studies with a total of 758 patients with drug-resistant epilepsy who underwent sEEG. The results showed similar depth error, radial error, EPE, TPE, pre-implantation time, number of electrodes per patient, and complication rates between robot-assisted and frame-based sEEG. However, robot-assisted sEEG had a significantly shorter overall operation time by 32.58 min in the total analysis and by 36.96 min in the skin-to-skin subgroup and a shorter operation time per electrode by 6.55 min. Data were heterogeneous for all variables except radial error, pre-implantation time, and complications. After addressing heterogeneity for EPE, TPE, overall operation time, and operation time per electrode, the initial findings were confirmed. However, the heterogeneity for the number of electrodes per patient remained unresolved. Interpretation of operation time per electrode should be tempered by the substantial and unresolved heterogeneity in electrode number per patient (I^2^ = 93%), which directly affects the denominator of this derived measure. Because operative time per electrode is a derived metric, variability in implantation strategy and electrode burden across studies limits its precision as a standalone efficiency outcome. Although multiple outcomes were assessed, the primary efficiency findings remained robust under conservative correction for multiple comparisons, supporting the reliability of these results.

The robotic technique enhances the efficiency of the setup and execution of sEEG. It facilitates the identification and localization of EZs, as well as the adjustment and repositioning of the electrode, while potentially reducing the need for repeated manual repositioning or repeated intraoperative verification steps, depending on institutional workflow and imaging protocols. Additionally, it allows a higher flexibility of the implant angle, expanding the reachable intracranial space. These features lend to a shorter overall operation time and operation time per electrode in robot-assisted sEEG. Shorter surgeries minimize anesthesia exposure for patients while reducing surgeon fatigue and the likelihood of subsequent errors.

Most authors supported the superiority of robot-assisted sEEG and its adaptability, especially for patients requiring more electrodes. We only analyzed overall intracranial hemorrhage and intracranial hematoma; although these differences did not reach statistical significance, there was a higher proportion of intracranial hematoma in the robot-assisted group (7.6% vs. 3.2%) and a comparable rate of intracranial hemorrhage (8.1% vs. 9%) compared with the frame-based group. Even if not statistically significant, certain hemorrhage subtypes appeared more frequent in the robot-assisted cohort, including intraparenchymal hemorrhage (11.76% vs. 5.55%), subdural hematoma (6.89% vs. 2.34%), and epidural hematoma (3.85% vs. 2.13%), whereas subarachnoid hemorrhage was only reported in the frame-based group (0% vs. 0.71%). Although these events were relatively rare, their presence highlights the importance of considering both the frequency and potential severity of hemorrhagic complications when comparing the safety profiles of robot-assisted and frame-based sEEG. The reason for this observed incidence in the robot-assisted cohort was not identified. Machetanz et al. [27] found a lack of relatedness between subdural hematoma in robot-assisted sEEG and EPE. The lower number of patients in the robot-assisted group might have underpowered the detection of significant differences.

The calculated heterogeneity for the operation time per electrode was eliminated after excluding Abdallat 2025 and Zheng 2021 [5, 47]; however, the heterogeneity for the overall operation time persisted even after excluding Abdallat 2025. This heterogeneity obstructs comparisons among studies. The operative time per electrode was calculated by dividing the operative time by the number of implanted electrodes. The data presented a broad variation in the number of electrodes per patient, ranging from 8.9 to 11.3 in robot-assisted sEEG and 6.4 to 12.56 in frame-based sEEG. The number of electrodes was larger in robot-assisted sEEG in some studies and frame-based sEEG in others, which might explain the high heterogeneity in overall operation time across studies.

Operative time and complication rates may also be influenced by the learning curve and institutional experience. In many centers, frame-based sEEG represents a long-established technique, whereas robot-assisted implantation was often adopted later. Consequently, early robot-assisted cases may reflect longer operative times or higher variability related to team familiarity, workflow optimization, and device-specific experience rather than intrinsic limitations of the robotic technique itself.

The primary goal of sEEG is to identify the EZ, which is the region of the brain where seizures originate. The number and placement of electrodes are determined by the location and number of EZs, as well as the results of noninvasive investigations, as described by Kahane et al. [23]. Several authors have proposed that the higher success achieved in increasing the number of implanted electrodes is due to the fact that it enhances the "ablative range," the coverage of the targeted area [17, 45]. Nevertheless, the implantation of intracerebral electrodes is associated with 1–8% morbidity, particularly hemorrhages. Kahane et al. [23] reported a 0.14–0.17% complication rate per electrode. The hemorrhage rate documented in the included studies ranged between 5–14.3% and 6.8–11.8% in frame-based sEEG and robot-assisted sEEG, respectively. The sEEG plan must account for the risk of increasing the number of electrodes, the complications associated with each approach, and adequate coverage of the epileptogenic network.

EPE is the difference between the actual and planned entry position of the electrode, which is affected by misregistration of the neuronavigation system, inaccurate alignment, and deflection during drilling. TPE is the difference between the actual and planned electrode positions at the target site, which is affected by the electrode entrance angle, deflection of the electrode, electrode rigidity, and depth of insertion. EPE and TPE are measured in several ways, with some, such as Euclidean distance, accounting for depth error. Variations among measurements contributed to the observed heterogeneity, which compromised comparability. A uniform rating scale is required to facilitate accurate comparison.

The robot-assisted method is recent, and surgeons have relatively limited experience with it. This could explain the reduced accuracy of the Autoguide robot in centers with early experience with it [20]. Fish et al. documented an improvement in accuracy with increased experience. The literature showed greater improvement in accuracy in the robot-assisted sEEG cohort. However, this improvement could be related to enhanced knowledge, experience, and surgical workflow rather than the device itself. Notably, some studies have advanced surgical workflows in robot-assisted sEEG, which might overestimate their accuracy. For instance, Yao et al. performed a micro-incision at the entry point before drilling, minimizing EPE [46]. The accuracy of implantation contributes to the accuracy of EZ localization, subsequent seizure freedom, and postoperative complications, particularly intracranial hemorrhage.

Determinants of accuracy include stability of registration, tapping power on the outer table at the start of drilling, drill type, firmness of the Mayfield clamp, epileptologist's experience, intracranial implant length, skull thickness, stability of the guidance screw, electrode entrance angle, and the electrode planning software used. Literature supports the major contribution of epileptologists' experience and the team's knowledge about anatomic-electrical-clinical factors to the success of implantation [16, 42, 46, 47]. In Zheng et al.'s study, the authors reported the achievement of Engel class I or II seizure outcomes. Implant planning and treatment options were decided through a discussion among multidisciplinary epilepsy specialists [47].

On the other hand, literature deviates regarding the direction of the correlation between skull thickness and the involvement of entry point accuracy in determining implant accuracy. While Desai et al. and Zheng et al. [5, 42] reported improved accuracy at thicker skull entry points, Sharma et al. associated increased soft tissue and bone thickness with reduced accuracy. Vakharia et al. [42] reported a displacement of the mechanical arm, causing greater target point and insertion angle errors in frameless sEEG insertion and iSYS1, the Autoguide precursor, with similar entry point accuracy. Discrepancies in the literature could arise from variations between and within implantation methods, reference processes, registration platforms, fiducial registration, imaging modalities, and surgery workflows. A meta-analysis reported the lowest EPE, TPE, and risk of complications in the iSYS1 robot compared with ROSA, Neuromate, and Sinovation robots [43].

## Limitations and future research recommendations

Our analysis was limited by the small number of included studies and their methodological differences, which restricted our ability to fully account for these variations and contributed to the high heterogeneity observed across studies. Nevertheless, performing a leave-one-out sensitivity analysis resolved heterogeneity for EPE, TPE, overall operation time, and operation time per electrode, thereby confirming the robustness of our primary findings. As emphasized in methodological literature, such analyses are exploratory and should not be interpreted as justification for post hoc study exclusion or heterogeneity resolution [33].

Although overall hemorrhagic complication rates were comparable between techniques, interpretation is limited by inconsistent reporting across studies. Given that sEEG is a diagnostic procedure with a low tolerance for neurological morbidity, the lack of standardized reporting of symptomatic versus incidental hemorrhage represents an important limitation of the existing literature. Our study results pertain to patients with drug-resistant epilepsy with a mean age of 26 years from China, France, Canada, Australia, Germany, and Taiwan. Furthermore, they are specific to the Leksell and Talairach frames, as well as ROSA, SINO, and Neuromate robots. Notably, the studies were retrospective and included adults, except for one study that involved children [6]. Large multicenter prospective and randomized controlled studies covering a broad range of age groups and all frame and robot systems are needed.

## Conclusion

This meta-analysis demonstrated that robot-assisted sEEG was associated with a significantly shorter overall operation duration and operation time per electrode compared with the frame-based group. Moreover, no significant differences were observed between the two groups in the pre-implantation time or the number of electrodes per patient. Both groups demonstrated comparable safety profiles, with no statistically significant differences in overall complications, including hemorrhagic events, neurological deficits, infections, or device-related technical issues; however, robot-assisted sEEG was associated with a minimally higher proportion of intraparenchymal hemorrhage and subdural hematoma. Further prospective studies with a wider range of robotic and frame-based systems are needed.

## Supplementary Information

Below is the link to the electronic supplementary material.

ESM 1 Supplementary Material 1 (DOCX 750 KB)

## Data Availability

Data were publicly available.

## Declarations

We confirm that this manuscript complies with all instructions to authors, that all authors meet the authorship criteria and have approved the final version of the manuscript, and the PRISMA checklist was used.

### Previous publication

We declare that this study has not been published at all.

Generative AI in scientific writing: We declare that we have not used AI in writing any part of this abstract.
