# Supplementary material for: Robot-assisted versus frame-based stereoelectroencephalography (sEEG) electrode implantation in drug-resistant epilepsy: a meta-analysis of accuracy, efficiency, and safety
Source: Acta Neurochir (Wien). 2026 Feb 21;168(1):46. doi: 10.1007/s00701-026-06787-6 (PMC12935791; doi:10.1007/s00701-026-06787-6)
Supplement: Supplementary file 1 — Supplementary Material 1 (DOCX 750 KB) [file 701_2026_6787_MOESM1_ESM.docx]

# Contents

[Contents	1](#_Toc220356433)

[**Supplementary Table 1. Bias Risk Assessment to Cohort Studies Using the Newcastle-Ottawa Scale Tool**	1](#_Toc220356434)

[**Supplementary Figure 1. Sensitivity Analysis of Entry Point Error (EPE)**	1](#_Toc220356435)

[**Supplementary Figure 2. Sensitivity Analysis of Target Point Error (TPE)**	2](#_Toc220356436)

[**Supplementary Figure 3. Sensitivity Analysis of Overall Operation Time (minutes)**	2](#_Toc220356437)

[**Supplementary Figure 4. Sensitivity Analysis of Operation Time per Electrode (minutes)**	2](#_Toc220356438)

[**Supplementary Figure 5. Publication Bias Assessment using the LFK Index for Outcomes with Five Studies or More**	3](#_Toc220356439)

# **Supplementary Table 1. Bias Risk Assessment to Cohort Studies Using the Newcastle-Ottawa Scale Tool**

| **Study** | **Selection** | | | | **Comparability** | **Outcome** | | | | **Total** |
| --- | --- | --- | --- | --- | --- | --- | --- | --- | --- | --- |
|  | **Representativeness of the Exposed Cohort** | **Selection of the Non-Exposed Cohort** | **Ascertainment of Exposure** | **Demonstration that Outcome Was Not Present at Baseline** |  | **Assessment of Outcome** | **Follow-Up Duration** | **Adequacy of Follow-Up** |  | |
| **Abdallat 2025** | * | * | * | * | * | * | * | * | 8/9 (Good) | |
| **Fish 2025** | 0 | * | * | * | 0 | * | * | * | 6/9 (Poor) | |
| **Han 2024** | * | * | * | * | * | * | * | * | 8/9 (Good) | |
| **Yao 2022** | * | * | * | * | * | * | * | * | 8/9 (Good) | |
| **Machetanz 2021** | * | * | * | * | * | * | * | * | 8/9 (Good) | |
| **Zheng 2021** | * | * | * | * | * | * | 0 | * | 7/9 (Good) | |
| **Bourdillon 2019** | * | * | * | * | * | * | * | * | 8/9 (Good) | |
| **Abel 2018** | * | * | * | * | * | * | * | * | 8/9 (Good) | |

The NOS tool reviews each observational study based on nine criteria that are organized into three groups: participant selection, group comparability, and the determination of either the exposure or outcome of interest. Each criterion is rated as either 'yes' or 'no'. Studies that achieve a score between 7 and 9 are classified as high quality, while those scoring between 5 and 6 are regarded as fair quality. Scores that fall between 1 and 4 are identified as poor quality.

# **Supplementary Figure 1. Sensitivity Analysis of Entry Point Error (EPE)**

Leave-one-out analysis excluding Han 2024 and Machetanz 2021 reduced heterogeneity and confirmed the non-significant pooled effect.

# **Supplementary Figure 2. Sensitivity Analysis of Target Point Error (TPE)**

Exclusion of Han 2024 reduced heterogeneity to 0%, with results remaining non-significant.

# **Supplementary Figure 3. Sensitivity Analysis of Overall Operation Time (minutes)**

Exclusion of Abdallat 2025 reduced heterogeneity while preserving the significant reduction in operation time with robot-assisted implantation.

# **Supplementary Figure 4. Sensitivity Analysis of Operation Time per Electrode (minutes)**

Exclusion of Abdallat 2025 and Zheng 2021 eliminated heterogeneity, confirming the robustness of the shorter operation time per electrode in the robot-assisted group.

# **Supplementary Figure 5. Publication Bias Assessment using the LFK Index for Outcomes with Five Studies or More**

Minor asymmetry was observed for EPE (LFK = −1.81) and TPE (LFK = −1.53). In contrast, major asymmetry was detected for overall operative time (LFK = −2.37), whereas no asymmetry was identified for the number of electrodes per patient (LFK = 0).

**EPE**

**TPE**

**Overall Operation Time**

**Number of Electrode per Patient**
